# Gene Therapy Restores *Mfrp* and Corrects Axial Eye Length

**DOI:** 10.1038/s41598-017-16275-8

**Published:** 2017-11-23

**Authors:** Gabriel Velez, Stephen H. Tsang, Yi-Ting Tsai, Chun-Wei Hsu, Anuradha Gore, Aliaa H. Abdelhakim, MaryAnn Mahajan, Ronald H. Silverman, Janet R. Sparrow, Alexander G. Bassuk, Vinit B. Mahajan

**Affiliations:** 1 Omics Laboratory, Stanford University, Palo Alto, CA USA; 2 Byers Eye Institute, Department of Ophthalmology, Stanford University, Palo Alto, CA USA; 3 Medical Scientist Training Program, University of Iowa, Iowa City, IA USA; 4 Bernard & Shirlee Brown Glaucoma Laboratory, Departments of Ophthalmology, Pathology and Cell Biology, Institute of Human Nutrition, Columbia University, New York, NY USA; 5 Edward S. Harkness Eye Institute, New York-Presbyterian Hospital, New York, NY USA; 6 Department of Pediatrics, University of Iowa, Iowa City, IA USA; 7 Department of Neurology, University of Iowa, Iowa City, IA USA; 8 Palo Alto Veterans Administration, Palo Alto, CA USA

## Abstract

Hyperopia (farsightedness) is a common and significant cause of visual impairment, and extreme hyperopia (nanophthalmos) is a consequence of loss-of-function *MFRP* mutations. *MFRP* deficiency causes abnormal eye growth along the visual axis and significant visual comorbidities, such as angle closure glaucoma, cystic macular edema, and exudative retinal detachment. The *Mfrp* ^*rd6*^ */Mfrp* ^*rd6*^ mouse is used as a pre-clinical animal model of retinal degeneration, and we found it was also hyperopic. To test the effect of restoring *Mfrp* expression, we delivered a wild-type *Mfrp* to the retinal pigmented epithelium (RPE) of *Mfrp* ^*rd6*^ */Mfrp* ^*rd6*^ mice via adeno-associated viral (AAV) gene therapy. Phenotypic rescue was evaluated using non-invasive, human clinical testing, including fundus auto-fluorescence, optical coherence tomography, electroretinography, and ultrasound. These analyses showed gene therapy restored retinal function and normalized axial length. Proteomic analysis of RPE tissue revealed rescue of specific proteins associated with eye growth and normal retinal and RPE function. The favorable response to gene therapy in *Mfrp* ^*rd6*^ */Mfrp* ^*rd6*^ mice suggests hyperopia and associated refractive errors may be amenable to AAV gene therapy.

## Introduction

Hyperopia (farsightedness) is a condition where distant objects can be seen more clearly than nearby ones; and an extreme form of hyperopia is caused by a rare, human genetic disorder known as nanophthalmos. Eyes of nanophthalmos patients are underdeveloped along the visual axis, causing the lens and cornea to be too close to the retina. Secondary complications are common because growth of a full-sized retina must be supported by tissues that only grow to cover less than half their normal area. This crowding in the eye leads to localized slippage between the retinal pigment epithelia (RPE) and the retina, causing deformations that further impair visual activity^1^. Serious complications can follow, such as angle closure glaucoma, cystic macular edema, and retinal detachment. Although the molecular mechanisms underlying hyperopia are poorly understood, gene therapy to correct a mutation that causes nanophthalmos (and extreme hyperopia) might correct the problem nonetheless. Such gene-therapy correction would have important implications not only for nanophthalmos but potentially also for ordinary cases of near- and farsightedness.

Mutations in human *MFRP (*membrane-type frizzled-related protein) gene cause hyperopia and nanophthalmos. Often, *MFRP-*deficient eyes have axial lengths ranging from 15.4 to 16.3 mm, relative to the population average of 23.5 mm, as well as spots of retinal discoloration and reduced electroretinogram (ERG) readings, brought about by the death of photoreceptors (a phenotype typical of nanophthalmos patients). These pathogenic changes may be reversible via gene therapy even without first determining how *MFRP* regulates eye length. *MFRP* is expressed in the retinal pigment epithelium; and previous studies showed the RPE regulates ocular growth^2^. Nanophthalmic eyes have a considerably thicker choroidal vascular bed and scleral coat, structures that provide nutritive and structural support for the retina. Thickening of these tissues is a general feature of axial hyperopia^1^. When hyperopia is experimentally induced by implanting myopic defocus lenses on the developing eyes of mice, they develop choroidal thickening, decreased scleral growth, and decreased vitreous chamber depth.

Modeling hyperopia in mice has been challenging, but r*d6* (*Mfrp* ^*rd6*^ */Mfrp* ^*rd6*^) mice could provide an important starting point. In these mice, a 4 bp deletion in the splice donor site of *Mfrp* exon 4 causes it to be skipped, deleting 58 residues from the MFRP protein. MFRP functions are highly eye-specific so in its absence otherwise healthy mice display white retinal spotting, photoreceptor death, and hyperopia^3^. This similarity to human disease makes it an ideal model to investigate therapeutic interventions and mechanisms underlying axial eye length. In this study, we tested whether *Mfrp* ^*rd6*^ */Mfrp* ^*rd6*^ mice can model hyperopia and whether gene therapy can rescue hyperopia. Using proteomic analysis of RPE-choroid tissue, we identified key proteins that were dysregulated in *Mfrp* ^*rd6*^ */Mfrp* ^*rd6*^ mice.

## Results

### *MFRP* mutations cause severe human hyperopia

A 5-year old boy was evaluated for posterior microphthalmos. His best-corrected visual acuity was 20/50-3 in the right eye and 20/60 in the left. Cycloplegic refraction revealed high hyperopia of +16.00 bilaterally. Ultrasound showed this was due to shortened eye axial lengths of 16.15 mm on the right, and 16.23 mm on the left (Fig. S1; Table 1). Indirect ophthalmoscopy detected retinal folds in the maculae in both eyes, a feature also detected by optical coherence tomography. There was no retinal pigmentary degeneration. Electroretinography revealed robust scotopic and photopic function, and Goldmann perimetry revealed normal visual fields, together confirming intact photoreceptor function. Genetic testing revealed a homozygous *MRFP* mutation (IVS10, +5, G > A) at the splice donor site of intron 10.

**Table 1:** Axial lengths and mutations of MFRP patients.

Case	Age	Sex	*MRFP* mutation	Axial length OD (mm)	Axial length OS (mm)
1	5	M	IVS10, +5, G > A, homozygous	16.15	16.23
2	61	F	+492, delC, homozygous	16.89	16.89
3	61	F	+492, delC, homozygous	17.26	16.96
4	19	M	Frameshift: c.1150dupC/p.His384Profs*8; Missense: c.1615C > T/p.Arg539Cys	15.00	15.06

**Figure 1:**
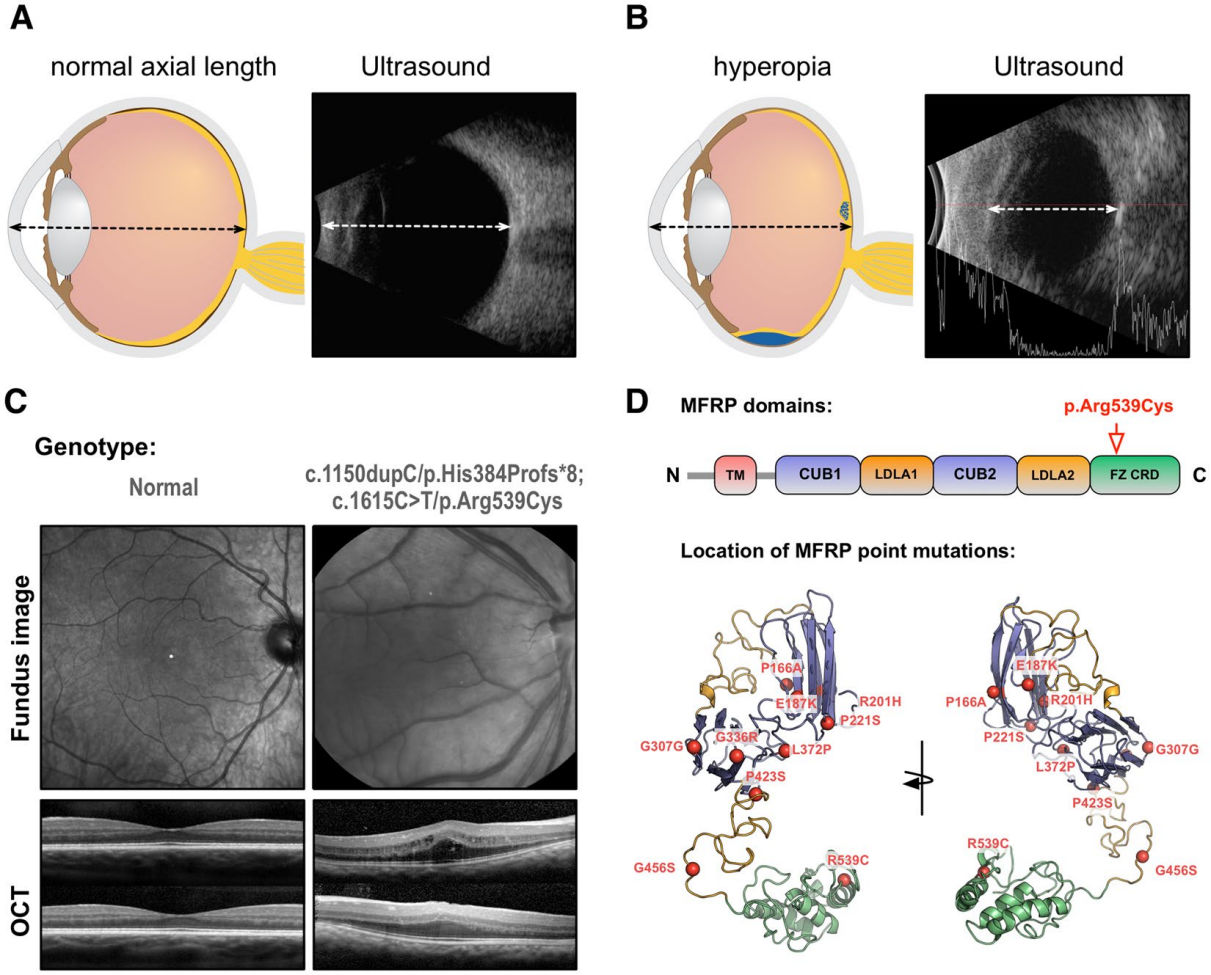
Human Phenotyping. (**A**) Schematic representation next to an ultrasound scan of a normal eye with normal axial length and a hyperopic eye with reduced axial length (**B**). Illustrations provided by Lucy Evans (acknowledgements section). (**C**) Clinical phenotype of a normal eye compared to that of a patient with *MFRP*-related hyperopia. Infra-red fundus photograph revealed no intra-retinal pigment migration. Optical coherence tomography (OCT) shows cystic degeneration and edema, but photoreceptor nuclei loss in the one of the twins with severe frameshift mutations. (**D**) Location of known *MFRP* point mutations span all domains in an MFRP structural model.

This clinical presentation emphasizes how, in early stages of the disease, *MFRP* patients suffer visual disability from their hyperopia that is distinct from any retinitis pigmentosa-like phenotype. In contrast, patients with typical retinitis pigmentosa preserve their central macular vision. In *MFRP* patients, however, the short axial eye length causes structural changes in the macula, such as macular folds, macular edema, and exudative retinal detachment. Thus, despite a physiologically functional macula, their shortened eye length causes macular degenerative changes later. For example, a 19-year old man with a *MRFP* mutation (frameshift: c.1150dupC/p.His384Profs*8; missense: c.1615C >T/p.Arg539Cys) had high hyperopia (+17.00) with shortened axial eye lengths (Fig. 1A–C). His macula had cystoid changes that reduced his vision to 20/80. Again, there was no intraretinal pigment migration and ERG testing revealed robust photopic function and residual scotopic function. Chronic degenerative changes generally do not develop until late stages of the disease. These late-stage changes are exemplified by two 61-year-old, female twins with an *MRFP* mutation (+492, delC, homozygous), who had severe hyperopia, and short axial lengths and eventually developed retinal degenerative changes in their maculae that reduced their vision to 20/400 (Fig. S1). Taken together, these clinical findings suggest that the hyperopic phenotype precedes the retinitis pigmentosa phenotype, and imply that early correction of the hyperopic phenotype could improve the secondary effect of short axial eye length, which is the primary contributor to functional vision loss.

### Gene therapy for *Mfrp*-related hyperopia

We performed structural modeling of known *MFRP* point mutations and found that they were localized across multiple functional domains (Fig. 1D; Figs S2–3; Table S1), lowering the likelihood of developing targeted small-molecule therapy and instead pointing to whole gene replacement. Gene therapy is used to treat human retinal degenerative disease^4^; and adenoviral vectors can be evaluated in murine preclinical models^5,6^. We previously showed that gene replacement therapy in *Mfrp* ^*rd6*^ */Mfrp* ^*rd6*^ mice reversed histological photoreceptor degeneration and normalized electrical retinal signaling^7^. While the patient *MFRP* mutations we identified were structurally-distinct from the *rd6* mutation, they are all predicted to result in loss-of-function, similar to the *Mfrp* ^*rd6*^ */Mfrp* ^*rd6*^ mouse model (Table S1). We performed sub-retinal injections of a viral vector carrying the normal mouse *Mfrp* gene. The vector was composed of the self-complementary Y733F tyrosine capsid mutant AAV2/8 (scAAV). The AAV2/8(Y733F)-*CBA*-*Mfrp* were sub-retinally injected into right (OD) eyes of *Mfrp* ^*rd6*^ */Mfrp* ^*rd6*^ mice at post-natal day 5 (P5). All left (OS) eyes, in both groups of mice, were maintained as matched controls for experimental analyses.

**Figure 2:**
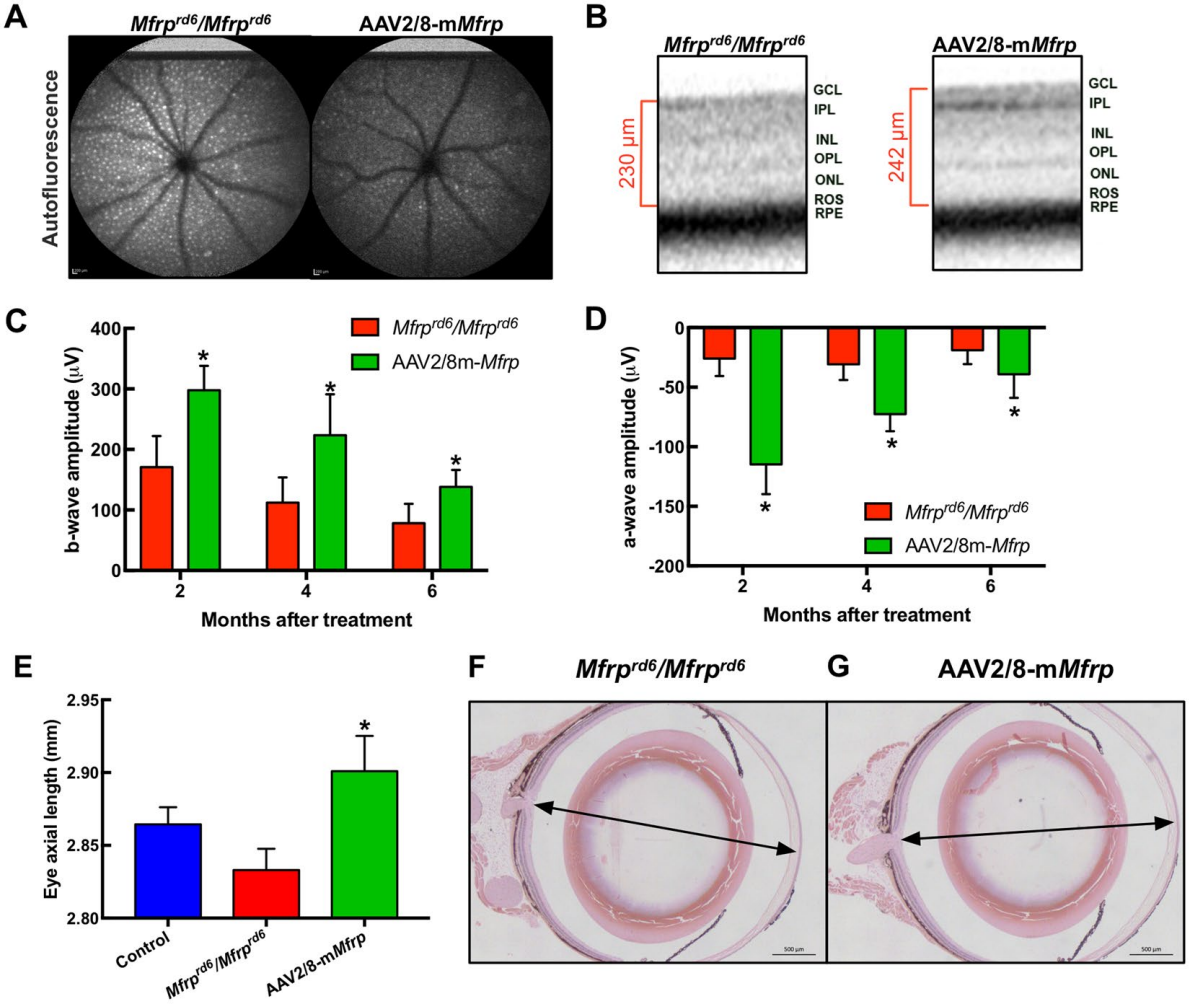
Retinal function and axial length recovery after AAV2/8-m*Mfrp* transduction. (**A**) Fundus autofluorescence (AF) of *Mfrp* ^*rd6*^ */Mfrp* ^*rd6*^ versus AAV2/8-m*Mfrp* treated eye. A fluorescence standard (an intensity comparison) appears as a bright strip at the top of each image. The AAV2/8-m*Mfrp* eye fluoresced with reduced AF intensity. (**B**) SD-OCT suggests rescue of retinal cell layers. Representative SD-OCT image shows cell layer thickness was 230 µm in the untreated eye and 242 µm in the treated eye. (**C**) Quantification of ERG b-wave amplitude shows a significant increase in retinal bipolar cell activity 2-months following gene therapy. Data were analyzed with a pairwise Student’s t-test (p = 0.04). (**D**) Quantification of ERG a-wave amplitude showing significant increase in photoreceptor cell function 2-months following gene therapy (p = 0.004). (**E**) Eye axial length was measured using the 50 MHz ultrasound bio-microscope (UBM) probe on an AVISO A/B (Quantel Medical) and reported in millimeters (8 eyes per group). Data were analyzed using 1-way ANOVA followed by Tukey’s multiple comparison test. AAV2/8-m*Mfrp* mice had a significant increase (average of 0.1 mm) in axial length compared to *Mfrp* ^*rd6*^ */Mfrp* ^*rd6*^ mice (p = 0.0334). Histological comparison of *Mfrp* ^*rd6*^ */Mfrp* ^*rd6*^ (**F**) and AAV2/8-m*Mfrp* mice (**G**) eye axial length.

To verify rescue of the *Mfrp* gene function, retinas were examined using non-invasive human clinical testing, which is highly translatable for human gene therapy (unlike histological analyses)^8^. Fundus autofluorescence imaging confirmed a reduction in hyper-fluorescent spots, indicating rescue of RPE function (Fig. 2A; p < 0.05). *In vivo* spectral domain OCT imaging also indicated retinal cell rescue (Fig. 2B). Electroretinography (ERG) confirmed rescue of photoreceptor function. Two months after sub-retinal injection of gene therapy vectors, mice showed significantly increased b-wave and reduced a-wave amplitudes, confirming the function of both rod and cone photoreceptors had improved (Fig. 2C,D; p < 0.05).

The degree of shortening necessary to produce distorted vision in mice has not been established, but in humans, even 1 mm of shortened length can lead to 20/400 vision (legally blind). Thus, in the much smaller mouse eye, even a small amount of distortion could account for a wide range of effects, from refractive errors to more serious conditions. In our mice, we extended eye length with our gene therapy injections, as shown by ultrasound (Fig. 2E; p < 0.05)^8^. Wild-type mice and control injection *Mfrp* ^*rd6*^ */Mfrp* ^*rd6*^ mice displayed normal and short eye lengths, respectively. Histological fixation of eyes confirmed these results (Fig. 2F,G). In total, gene therapy restored axial length, along with auto-fluorescence and photoreceptor survival in the *Mfrp* ^*rd6*^ */Mfrp* ^*rd6*^ mice.

### Proteomic analysis of RPE-choroid signaling pathways

**Figure 3:**
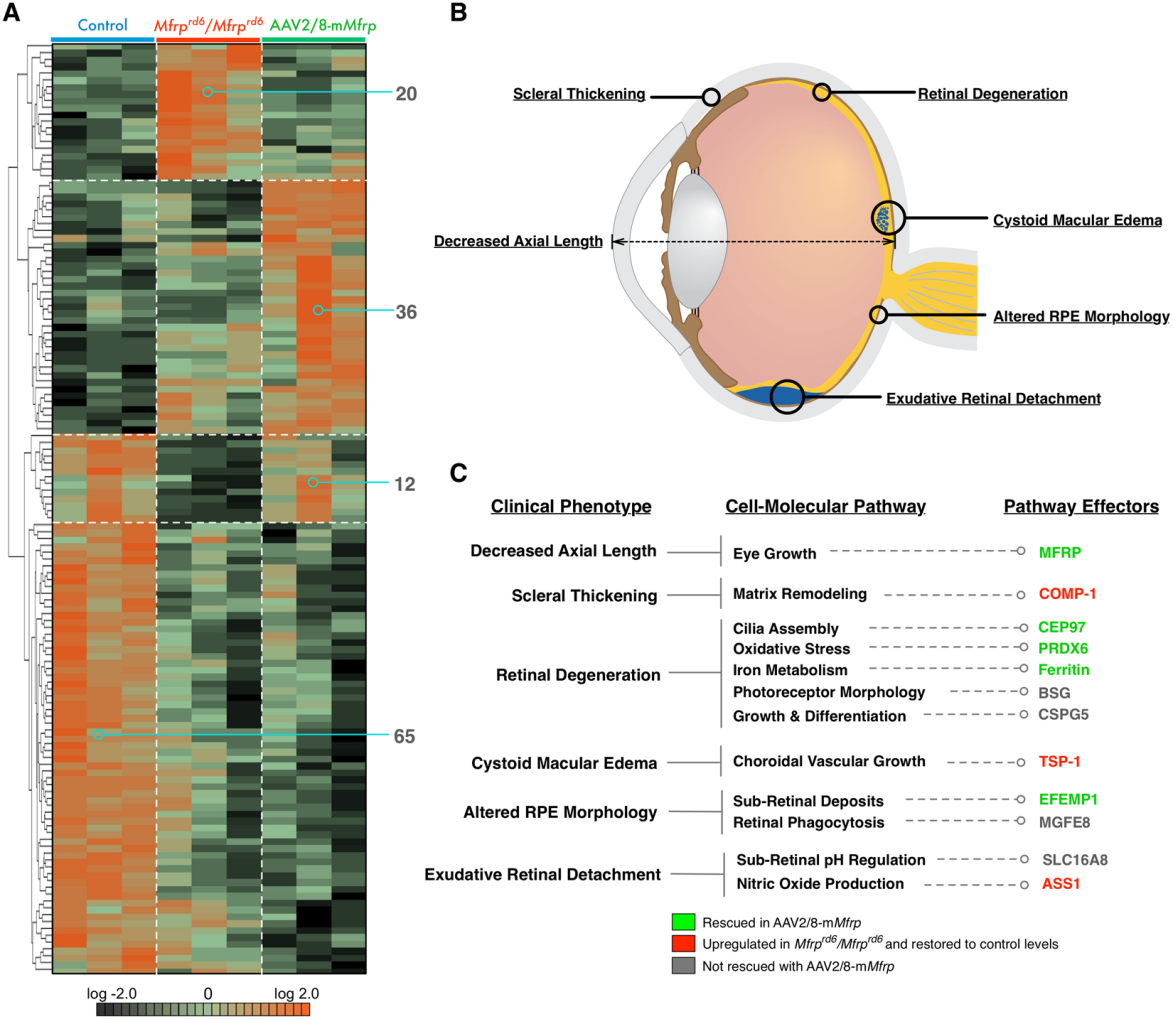
Proteomic analysis of *Mfrp* ^*rd6*^ */Mfrp* ^*rd6*^ and AAV2/8-m*Mfrp* RPE reveal differentially-expressed proteins. (**A**) Hierarchal clustering of proteins differentially-expressed in *Mfrp* ^*rd6*^ */Mfrp* ^*rd6*^ and AAV2/8-m*Mfrp* mice compared to B6 controls. Results are represented as a heatmap and display protein levels on a logarithmic scale. A total of 137 proteins were differentially-expressed among the 3 groups (p < 0.05). (**B**) Pathogenic features of nanophthalmos. Illustration provided by Lucy Evans (acknowledgements section). (**C**) Correlations between pathogenic features of high hyperopia and the molecular pathways identified in our proteomic analysis.

Previously, gene therapy in our *Mfrp* ^*rd6*^ */Mfrp* ^*rd6*^ mice restored the normal hexagonal morphology of RPE cells^7^. We dissected the RPE-choroid tissue from control (C57BL/6), *Mfrp* ^*rd6*^ */Mfrp* ^*rd6*^, and AAV2/8-m*Mfrp* mice, and analyzed proteomic content via liquid chromatography tandem mass spectrometry (Fig. S4). Protein intensity data were analyzed with 1-way ANOVA and unbiased hierarchical clustering. A total of 137 proteins were differentially-expressed among the three groups (Fig. 3A; p < 0.05). Based on the hierarchal clustering, proteins were grouped into four categories: proteins upregulated in *Mfrp* ^*rd6*^ */Mfrp* ^*rd6*^ mice (Table S2), proteins upregulated in response to AAV injection (Table S3), proteins rescued following gene therapy (Table S4), and proteins not rescued (Table S5). These proteins were queried using pathway analysis (Fig. S5). As a control, we identified 36 proteins upregulated in AAV2/8-m*Mfrp* RPE compared to C57BL/6 (Fig. 3A). These proteins were not present at significant levels in controls, suggesting they were not rescued, but rather expressed as a response to AAV injection. Since sub-retinal injection of AAV2 vectors can induce expression of signaling pathways, we controlled for proteins by subtracting them from our ‘rescued proteins’ list (Table S3; Fig. S5)^9^.

*Mfrp* ^*rd6*^ */Mfrp* ^*rd6*^ mice display photoreceptor cell death and progressive retinal dysfunction. We identified downregulation of protein pathways linked to retinal degeneration: cilia assembly (CEP97), oxidative stress (PRDX6), iron metabolism (Ferritin), and cell growth (BSG and CSPG5; Fig. 3B,C)^10–15^. Shed rod outer segments accumulate in the sub-retinal space of *Mfrp* ^*rd6*^ */Mfrp* ^*rd6*^ mice^3,16^. This suggests decreased retinal phagocytosis following loss of *Mfrp* function. Decreased retinal phagocytosis is implicated as a cause of age-related blindness^17^. MFGE8, a protein involved in retinal phagocytosis, was downregulated in *Mfrp* ^*rd6*^ */Mfrp* ^*rd6*^ RPE, suggesting that MFRP signaling may be upstream of this process (Fig. 3C). In our previous study, we showed that *Mfrp* ^*rd6*^ */Mfrp* ^*rd6*^ mice develop altered RPE morphology, which was reversed by gene therapy (Fig. S3). Levels of EFEMP1, a protein mutated in Malattia Leventinese (MLVT), were rescued following gene therapy. MLVT patients develop retinal degeneration and sub-retinal deposits like *Mfrp* ^*rd6*^ */Mfrp* ^*rd6*^ mice^18,19^. This rescue of EFEMP1 may explain our previous rescue of RPE morphology in *Mfrp* ^*rd6*^ */Mfrp* ^*rd6*^ mice.

Our proteomic analysis gave insight into other pathologic features of *MFRP*-related nanophthalmos. Patients develop scleral thickening, cystoid macular edema, and exudative retinal detachment (Fig. 3B). We identified proteins associated with extracellular matrix remodeling (e.g. COMP-1) in *Mfrp* ^*rd6*^ */Mfrp* ^*rd6*^ mice^20^. This may explain the scleral thickening phenotype. TSP-1 was also highly expressed in *Mfrp* ^*rd6*^ */Mfrp* ^*rd6*^ RPE. TSP-1 plays a role in regulating choroidal vascular permeability, which may contribute to cystoid macular edema (Fig. 3C)^21^. We noticed high argininosuccinate synthase-1 (ASS1) levels. ASS1 promotes the formation of nitric oxide, which causes dilation of the retinal vasculature, promoting increased vascular leakage that may cause exudative retinal detachment^22^.

## Discussion

The retinal degeneration phenotype of *Mfrp* ^*rd6*^ */Mfrp* ^*rd6*^ mice has been well-described: mice display white retinal spotting and photoreceptor degeneration^7^. However, descriptions of decreased axial length in these mice have not been described in the literature^3,16^. To date, there have been no regenerative medicine approaches that could reverse hyperopia in mice. A 1-mm change in the normal 24-mm axial length of human eyes is sufficient to significantly reduce visual acuity. Similar axial length changes in the mouse globe (2.8-mm average in C57BL/6 mice) would also cause significant refractive error^23^. Our study found an average 0.1-mm improvement in *Mfrp* ^*rd6*^ */Mfrp* ^*rd6*^ mouse eyes treated with gene therapy, which is a fractional change sufficient to produce vision improvement in human eyes. AAV2/8(Y733F)-*CBA*-*Mfrp* injections were found to rescue photoreceptor death, normalize retina function, reduce signs of retinal damage, and regulate eye length in adult mice (Fig. 2). These findings are promising in terms of treating the human diseases caused by *MFRP* mutations. Delivery of gene therapy vectors to the RPE is feasible and could influence eye growth^24^. With continued successful implementation of ocular gene therapy in patients, *MFRP*-related nanophthalmos may soon be treatable by gene therapy.

The RPE is thought to play a role in secreting signal molecules to the choroid. Previous studies have exemplified this by showing that imposed myopic defocus (inducing hyperopia) resulted in altered gene expression in the RPE and choroid^25^. Several molecular pathways are implicated in the development of small eyes, such as Smad4-mediated regulation of retinal Hedgehog and Wnt signaling^26^. However, the molecular mechanisms of nanophthalmos are poorly understood^27^. To this end, we used proteomic analysis of RPE-choroid tissue from *Mfrp* ^*rd6*^ */Mfrp* ^*rd6*^ and AAV-transduced mice to determine differentially-expressed proteins in our mouse model. Using these results, we created a model of molecular pathways affected in *MFRP*-related nanophthalmos (Fig. 3C). While *MFRP* mutations have been linked to nanophthalmos and microphthalmia, the function of the MFRP protein is unknown. MFRP contains functional domains that are related to signal transduction, proteolysis, and endocytosis^1^. The function of these domains and the molecular pathways downstream of MFRP signaling have not been established. Our proteomic analysis allowed us to identify protein pathways that are dysregulated following loss of *Mfrp* function. These affected pathways provide initial insight into the functions of MFRP beyond the regulation of eye growth.

## Methods

### Study approval

This study was approved by the Institutional Review Board for Human Subjects Research at Columbia University, was compliant with the Health Insurance Portability and Accountability Act, and adhered to the tenets of the Declaration of Helsinki (IRB Protocol AAAF1849). Written informed consent was received from participants prior to inclusion in the study. Color fundus pictures, optical coherence tomography (OCT), and electroretinogram (ERG) analysis were performed on four *MFRP*-related nanophthalmos patients in the Department of Ophthalmology, Columbia University Medical Center/New York Presbyterian Hospital.

#### Ethics Statement

The mouse procedures were approved by the Institutional Animal Care and Use Committee of Columbia University. Mice were used in accordance with the Statement for the Use of Animals issued by the Association for Research in Vision and Ophthalmology, as well as the Policy for the Use of Animals in Neuroscience Research of the Society for Neuroscience.

### Phenotypic Ascertainment in humans

The collection of data used in this study was approved by the Institutional Review Board for Human Subjects Research at Columbia University Medical Center, was compliant with the Health Insurance Portability and Accountability Act, and adhered to the tenets of the Declaration of Helsinki. Written informed consent was received from participants prior to inclusion in the study. Clinical examination and testing and genetic testing was performed as previously described^28^. Spectral domain optical coherence tomography (SD-OCT) were obtained using Spectralis Heidelberg (Heidelberg, Germany). Genomic DNA from patients was isolated from peripheral blood lymphocytes per standard methods. The entire coding sequence and exon-intron boundaries of *MFRP* (13 exons) were amplified by PCR using pairs of primers that were designed based on the published consensus sequences. Direct sequencing of the PCR-amplified products was analyzed by the Genwize Company (NJ). All *MFRP* exon data are available at Gene Expression Omnibus (MIM 606227).

### Homology modeling of human *MFRP*

We modeled the MFRP structure using a domain assembly approach: homology models of the cubulin domains (CUB1 and CUB2) were generated using the crystal structure of cubulin (PDB: 3KQ4) as a template with MODELLER 9.14, as described previously^28–31^. The LDLA1 and LDLA2 domains were generated based off the LDLR structure (PDB: 3P5B). Finally, the frizzled domain (FZ) was modelled using the XWnt8-bound Frizzled-8 structure (PDB: 4F0A) as a template. The individual domains were joined using the homology-modeling protocol in the YASARA 15.7.25 software package to generate a MFRP model. The model was refined with an energy minimization in the YAMBER3^32^ force field followed by a steepest descent minimization and simulated annealing.

### Mouse lines and husbandry

*Mfrp* ^*rd6*^ */Mfrp* ^*rd6*^ and C57BL/6 mice were bred and maintained at the facilities of Columbia University. Animals were housed individually and kept on a light–dark cycle (12 hour–12 hour) before the experiment. Food and water were available *ad libitum*.

### Adeno-associated virus (AAV) preparation and transduction into mice

Vectors were constructed and then sent to Penn Vector Corporation for production as previously described^7^. A total of 1 μl of AAV2/8(Y733F)::m*Mfrp* (1.23e^12^ genome copy/ml) was transduced into the subretinal space of the right eye of *Mfrp* ^*rd6*^ */Mfrp* ^*rd6*^ mice at postnatal day 5, which caused an ideal bleb detachment at the retinal site of the injection. The left eyes of all mice were left untouched and maintained as a control for experimental analyses. Anesthesia and surgery were performed as previously described^7^.

### Non-invasive imaging

*Mfrp* ^*rd6*^ */Mfrp* ^*rd6*^ (n = 3) and AAV2/8-m*Mfrp* mice (n = 3) were anesthetized as previously described using intraperitoneal ketamine (100 mg/kg) and xylazine (10 mg/kg) injections^7^. Pupils were dilated to a mean diameter of 2.5 mm with 1% tropicamide and 2.5% phenylephrine 15 minutes before image acquisition. The fundus was aligned as previously described and the retina was pre-exposed at 488 nm in auto-fluorescence mode for 20 seconds to bleach the visual pigment. Detector sensitivity was set at an optimal range that was then used for all auto-fluorescence imaging. Retinas were imaged by SD-OCT using a Heidelberg Spectralis HRA + OCT system (Heidelberg Engineering, Heidelberg, Germany). Non-correcting contact lenses to prevent corneal desiccation. SD-OCT was taken as horizontal line scan though the central retina 0.5 mm from the optic nerve. Dark-adapted ERGs were elicited with 0.02 and 2 scot-cd.s.m^−2^ stimuli. Espion ERG Diagnosys equipment (Diagnosys LLC, Lowell, MA) was used for the recordings. Eye axial length was measured using the 50 MHz ultrasound bio-microscope (UBM) probe on an AVISO A/B (Quantel Medical, Bozeman, MT).

### Histology of AAV transduced eyes

After subretinal injection of AAV2/8-m*Mfrp*, *Mfrp* ^*rd6*^ */Mfrp* ^*rd6*^ mice were sacrificed. Eyes were enucleated and fixed in 1:2x Karnovsky fixative (2% Paraformaldehyde and 1.5% glutaraldehyde) for 24 hours as previously described^7^. Eyes were embedded in paraffin, sectioned, and stained with hematoxylin and eosin before being visualized by light microscopy (Leica DM 5000B, Leica Microsystems, Germany).

### Proteomic analysis

Proteins were extracted from mouse RPE, precipitated in chloroform-methanol, and dissolved in 0.1% Rapigest detergent in 50 mM ammonium bicarbonate. Shotgun proteomic mass spectrometry-based measurements were performed in triplicate on *Mfrp* ^*rd6*^ */Mfrp* ^*rd6*^, AAV2/8::m*Mfrp*, and B6 control RPE. A liquid chromatography-tandem mass spectrometry (LC-MS/MS) approach was used for the relative quantitation and simultaneous identification of proteins from all three samples, including triplicate LC/MS/MS chromatograms (technical replicates) collected for each of the three biological replicates. We used Synapt G2 quadrupole-time-of-flight mass spectrometry (QTOF; Waters Corporation, Milford, MA). The data were analyzed with MS^E^/Identity^E^ algorithm (PLGS software Version 2.5 RC9) and Rosetta Elucidator software. Elucidator software detected 383,353 features across 27 LC/MS runs. Identifications were returned on 3,132 proteins with a PLGS score >300 (pass 1 data only) and 4% false discovery rate. Of the 3,132 proteins, 2,089 were represented by two or more peptides and used in further consideration and analysis. Of those 2,089 proteins, there were no missing values among the 27 LC/MS runs (56,403 protein expression quantitative determinations).

### Statistical and Bioinformatics analysis

Results were also saved in Excel as.txt format and were uploaded into the Partek Genomics Suite 6.5 software package as previously described^33–37^. The protein intensity data were normalized to log base 2, and compared using 1-way ANOVA analysis. All proteins with non-significant (p > 0.05) changes were eliminated from the table. The significant values were mapped using the ‘cluster based on significant genes’ visualization function with the standardization option chosen. Reactome Pathway Analysis^38^ was utilized to determine the most significant cellular pathways affected by the proteins present in *Mfrp* ^*rd6*^ */Mfrp* ^*rd6*^, AAV2/8-m*Mfrp*, and B6 control mice RPE.

### Data availability

The datasets generated during and/or analyzed during the current study are available from the corresponding author on reasonable request.

## Electronic supplementary material

Supplementary Information
