# Supplementary material for: Gene Therapy Restores Mfrp and Corrects Axial Eye Length
Source: Sci Rep. 2017 Nov 23;7:16151. doi: 10.1038/s41598-017-16275-8 (PMC5701072; doi:10.1038/s41598-017-16275-8)
Supplement: Supplementary file 1 — Supplementary Information [file 41598_2017_16275_MOESM1_ESM.pdf]

SUPPLEMENTARY ONLINE CONTENT

Gene Therapy Restores *Mfrp* and Corrects Eye Axial Length

Gabriel Velez, Stephen H. Tsang, Yi-Ting Tsai, Chun-Wei Hsu, Anuradha Gore, Aliaa H. Abdelhakim, MaryAnn Mahajan, Ronald H Silverman, Janet R. Sparrow, Alexander G. Bassuk, Vinit B. Mahajan.

Table of Contents

- **Supplemental Results**
- **Supplemental References**
- **Supplemental Figure 1.** Clinical Phenotype of Additional *MFRP* Patients
- **Supplemental Figure 2.** Individual MFRP Domain Models
- **Supplemental Figure 3.** Structural Modeling of MFRP Mutations
- **Supplemental Figure 4.** RPE Proteomics Pipeline
- **Supplemental Figure 5.** Pathway Analysis
- **Supplemental Table 1.** *MFRP* Point Mutations
- **Supplemental Table 2.** Proteins upregulated in $Mfrp^{rd6}/Mfrp^{rd6}$ mice
- **Supplemental Table 3.** Proteins upregulated as a response to AAV injection
- **Supplemental Table 4.** Proteins rescued by gene therapy
- **Supplemental Table 5.** Proteins not rescued by gene therapy

Supplemental Results

Structural modeling of human MFRP mutations

It is not known how MFRP mutations cause nanophthalmos or other retinal diseases, its exact role in the cell unknown, and its crystal structure is unsolved, so to gain insight into how point mutations might affect MFRP function, we used structural modeling. This analysis predicted several domains related to signal transduction, proteolysis, and endocytosis: two globular cubulin domains (CUB1 and CUB2), two domains related to the low-density lipoprotein receptor (LDLA1 and LDLA2), and a frizzled cysteine-rich domain (CRD). Further, the MFRP N-terminal region contains a putative transmembrane domain (TM; residues 70-90), suggesting it is a type II transmembrane protein with its C-terminal region projecting out to the extracellular space.[1]

In the absence of a high-resolution structure for MFRP, we used structural modeling to ascertain how our patient's mutations might alter MFRP structure and function.[2] We also predicted how other *MFRP* mutations reported in the published literature might affect predicted functions of MFRP (Table S1). MFRP, is composed of 4 distinct domains, so we used a domain-assembly strategy to model MFRP structure, based on homology to related structures: cubulin domains (CUB1 and CUB2) were modeled using the crystal structure of cubulin as a template; LDLA1 and LDLA2 domains were generated based on the LDLR structure; the CRD domain was modelled based on the XWnt8-bound Frizzled-8 structure (Fig. S2A-E). These individual domains were then assembled to generate a full-length MFRP model (Fig. S3).

Using this complete MFRP model, the mutations were mapped and found in all four domains (Fig. S3), allowing predictions as to how the mutations might affect protein function. For example, cubilin (CUB) domains are extracellular motifs comprised of ten β-strands in two five-stranded β-sheets [3] that mediate protein-ligand interactions and proteolysis, like in the Tolloid proteinase family (e.g. BMP-1).[3,4] Thus, although MFRP's catalytic activity has not been demonstrated, the presence of Tolloid repeats (CUB and LDLA domains) implicate MFRP in proteolytic pathways. Mutations in these domains could affect catalytic activity (Table 2). Likewise, MFRP's CRD contains a 40% identity to the CRDs present on SFRPs and Frizzled receptors. The CRD domain is a cysteine-rich motif in secreted frizzled receptor proteins (SFRPs) and frizzled receptors, and bind Wnt and regulate Wnt-signaling pathways. [5] Although interactions between MFRP and Wnt have not been demonstrated, its role in cell fate signaling is suggested by the presence of the CRD domain.[5,6] These predictions notwithstanding, since the mutations are scattered throughout several different functional domains, small-molecule therapy or highly targeted gene editing may not be feasible (Fig. S3; Table S1). Instead, either a cell-based therapy or gene replacement therapy might be more effective.

Proteomic Analysis

We first identified 20 proteins that were significantly upregulated in *Mfrp*^{rd6}/*Mfrp*^{rd6}. Among these proteins were thrombospondin-1 (TSP-1), argininosuccinate synthase-1 (ASS1), and cartilage oligomeric protein-1 (COMP-1). TSP-1 is a pro-angiogenic factor produced by the RPE and is believed to modulate choroidal vascular growth.[7] Increased TSP-1 levels may explain the vascular leakage that contributes to cystoid macular edema

in nanophthalmos patients (Fig. 1C). ASS1 is involved in the production of arginine and nitric oxide (NO). NO is a potent vasodilator in the central nervous system and retina.[8] We hypothesized that increased retinal vasodilation would lead to increased intravascular leakage, causing exudative retinal detachment in nanophthalmos patients.[9] COMP-1 is an extracellular matrix protein and a marker of collagen turnover.[10] Increased levels of COMP-1 may promote scleral thickening, one of the pathological features of nanophthalmos.[11] Interestingly, this protein was one of the most prominently expressed proteins in *Mfrp*^{rd6}/*Mfrp*^{rd6} mice. These proteins returned to control levels following gene therapy (Fig. 3A; Fig S4A).

We then identified 12 proteins were downregulated in *Mfrp*^{rd6}/*Mfrp*^{rd6} compared to controls and then restored to control levels following AAV2/8-m*Mfrp* injection. Among these proteins were EGF-containing fibulin-like extracellular matrix protein 1 (EFEMP1), peroredoxin 6 (PRDX6), centrosomal protein of 97 kDa (CEP97), and ferritin (Table S4). Interestingly, mutations in *EFEMP1* are known to cause a Mendelian form of macular degeneration known as Malattia Leventinese (MLVT). Patients with MLVT often display altered RPE cell ultrastructure, sub-retinal RPE deposits, and separation between the RPE and Bruch's membrane.[12,13] Notably, our *Mfrp*^{rd6}/*Mfrp*^{rd6} mice displayed similarly altered RPE morphology in our RPE wet mount, suggesting that rescue of EFEMP1 levels contributed to restored RPE morphology (Fig. S3).[14] PRDX6 is an antioxidant protein expressed in astrocytes and Müller cells. Since oxidative stress is implicated in retinal degeneration, we hypothesize that restoration of PRDX6 levels in the RPE contributed to reduced photoreceptor cell death and normalized retinal function in AAV2/8-m*Mfrp* mice.[15,16] CEP97 is involved in regulating cilia assembly, a process that is critical to the maintenance of photoreceptor outer segments in the retina.[17] Finally, ferritin is involved in iron storage and transport. Increased iron levels in the eye have been implicated in oxidative stress and retinal degeneration.[18] Gene therapy rescue of ferritin may play a role in restoring iron levels in the retina and decreasing toxicity.

Finally, we identified 65 proteins that were not rescued following gene therapy. Among these proteins were lacadherin (MFGE8), basigin (BSG), monocarboxylate transporter 3 (SLC16A8), and chondroitin sulfate proteoglycan 5 (CSPG5; Table S4). MFGE8 is involved in regulating diurnal phagocytosis of photoreceptor outer segments by the RPE.[19] Lack of synchronized retinal phagocytosis is implicated in age-related blindness. BSG is a glycoprotein that is implicated in retinal development. Knockout of *Bsg* in mice has been shown to cause altered retinal function, photoreceptor degeneration, and choroidal neovascularization.[20,21] SLC16A8 is a proton-coupled monocarboxylate transporter involved in sub-retinal pH regulation. Interestingly, knockout of *Scl16a8* in mice leads to a decrease in subretinal pH due to the accumulation of lactate and causes altered visual function.[22] Previous genome-wide association studies (GWAS) have implicated the *SLC16A8* locus in age-related macular degeneration.[23] CSPG5 is a chondroitin-sulfate proteoglycan that has been previously found to be upregulated in mouse models of retinal degeneration (*Rpe65*$^{-/-}$). Although increased levels of CSPG5 have been implicated in retinal degeneration, its role in this process is poorly understood.[24]

Supplemental References

1 Katoh, M. Molecular cloning and characterization of MFRP, a novel gene encoding a membrane-type Frizzled-related protein. *Biochem. Biophys. Res. Commun.* **282**, 116-123, doi:10.1006/bbrc.2001.4551 (2001).

2 Moshfegh, Y. *et al.* BESTROPHIN1 mutations cause defective chloride conductance in patient stem cell-derived RPE. *Hum. Mol. Genet.* **25**, 2672-2680, doi:10.1093/hmg/ddw126 (2016).

3 Gaboriaud, C. *et al.* Structure and properties of the Ca(2+)-binding CUB domain, a widespread ligand-recognition unit involved in major biological functions. *Biochem. J.* **439**, 185-193, doi:10.1042/BJ20111027 (2011).

4 Hartigan, N., Garrigue-Antar, L. & Kadler, K. E. Bone morphogenetic protein-1 (BMP-1). Identification of the minimal domain structure for procollagen C-proteinase activity. *J. Biol. Chem.* **278**, 18045-18049, doi:10.1074/jbc.M211448200 (2003).

5 Jones, S. E. & Jomary, C. Secreted Frizzled-related proteins: searching for relationships and patterns. *Bioessays* **24**, 811-820, doi:10.1002/bies.10136 (2002).

6 Sundin, O. H. *et al.* Extreme hyperopia is the result of null mutations in MFRP, which encodes a Frizzled-related protein. *Proc. Natl. Acad. Sci. U. S. A.* **102**, 9553-9558, doi:10.1073/pnas.0501451102 (2005).

7 Miyajima-Uchida, H. *et al.* Production and accumulation of thrombospondin-1 in human retinal pigment epithelial cells. *Invest. Ophthalmol. Vis. Sci.* **41**, 561-567 (2000).

8 Goldstein, I. M., Ostwald, P. & Roth, S. Nitric oxide: a review of its role in retinal function and disease. *Vision Res.* **36**, 2979-2994 (1996).

9 Bach, A. *et al.* Spontaneous exudative retinal detachment in a patient with sturge-weber syndrome after taking arginine, a supplement for erectile dysfunction. *Eye Vis (Lond)* **1**, 7, doi:10.1186/s40662-014-0007-x (2014).

10 Saxne, T. & Heinegard, D. Cartilage oligomeric matrix protein: a novel marker of cartilage turnover detectable in synovial fluid and blood. *Br. J. Rheumatol.* **31**, 583-591 (1992).

11 Stewart, D. H., 3rd *et al.* Abnormal scleral collagen in nanophthalmos. An ultrastructural study. *Arch. Ophthalmol.* **109**, 1017-1025 (1991).

12 Marmorstein, L. Y., McLaughlin, P. J., Peachey, N. S., Sasaki, T. & Marmorstein, A. D. Formation and progression of sub-retinal pigment epithelium deposits in Efemp1 mutation knock-in mice: a model for the early pathogenic course of macular degeneration. *Hum. Mol. Genet.* **16**, 2423-2432, doi:10.1093/hmg/ddm199 (2007).

13 Gerth, C., Zawadzki, R. J., Werner, J. S. & Heon, E. Retinal microstructure in patients with EFEMP1 retinal dystrophy evaluated by Fourier domain OCT. *Eye (Lond.)* **23**, 480-483, doi:10.1038/eye.2008.251 (2009).

14 Li, Y. *et al.* Gene therapy in patient-specific stem cell lines and a preclinical model of retinitis pigmentosa with membrane frizzled-related protein defects. *Mol. Ther.* **22**, 1688-1697, doi:10.1038/mt.2014.100 (2014).

15 Beatty, S., Koh, H., Phil, M., Henson, D. & Boulton, M. The role of oxidative stress in the pathogenesis of age-related macular degeneration. *Surv. Ophthalmol.* **45**, 115-134 (2000).

16 Chidlow, G., Wood, J. P., Knoops, B. & Casson, R. J. Expression and distribution of peroxiredoxins in the retina and optic nerve. *Brain Struct Funct* **221**, 3903-3925, doi:10.1007/s00429-015-1135-3 (2016).

17 Spektor, A., Tsang, W. Y., Khoo, D. & Dynlacht, B. D. Cep97 and CP110 suppress a cilia assembly program. *Cell* **130**, 678-690, doi:10.1016/j.cell.2007.06.027 (2007).

18 Wong, R. W., Richa, D. C., Hahn, P., Green, W. R. & Dunaief, J. L. Iron toxicity as a potential factor in AMD. *Retina* **27**, 997-1003, doi:10.1097/IAE.0b013e318074c290 (2007).

19 Nandrot, E. F. & Finnemann, S. C. Lack of alphavbeta5 integrin receptor or its ligand MFG-E8: distinct effects on retinal function. *Ophthalmic Res.* **40**, 120-123, doi:10.1159/000119861 (2008).

20 Hori, K. *et al.* Retinal dysfunction in basigin deficiency. *Invest. Ophthalmol. Vis. Sci.* **41**, 3128-3133 (2000).

21 Ochrietor, J. D. & Linser, P. J. 5A11/Basigin gene products are necessary for proper maturation and function of the retina. *Dev. Neurosci.* **26**, 380-387, doi:10.1159/000082280 (2004).

22 Daniele, L. L., Sauer, B., Gallagher, S. M., Pugh, E. N., Jr. & Philp, N. J. Altered visual function in monocarboxylate transporter 3 (Slc16a8) knockout mice. *Am. J. Physiol. Cell Physiol.* **295**, C451-457, doi:10.1152/ajpcell.00124.2008 (2008).

23 Fritsche, L. G. *et al.* Seven new loci associated with age-related macular degeneration. *Nat. Genet.* **45**, 433-439, 439e431-432, doi:10.1038/ng.2578 (2013).

24 Cottet, S. *et al.* Retinal pigment epithelium protein of 65 kDA gene-linked retinal degeneration is not modulated by chicken acidic leucine-rich epidermal growth factor-like domain containing brain protein/Neuroglycan C/ chondroitin sulfate proteoglycan 5. *Mol. Vis.* **19**, 2312-2320 (2013).

Supplementary Figure 1. Fundus images and SD-OCT analysis of two patients with *MFRP* mutations.

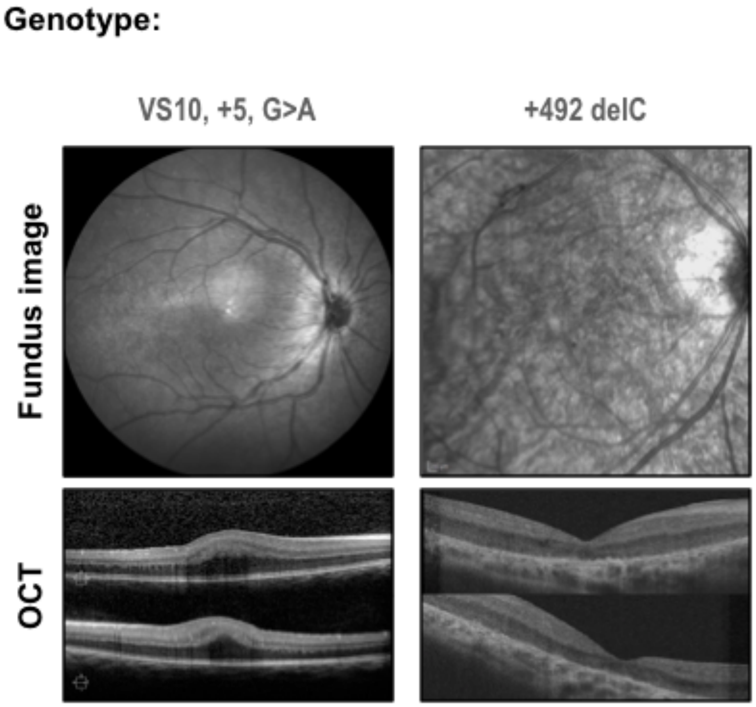

Supplementary Figure 2. Individual MFRP Domain Models. (A) MFRP CUB1 domain modeled off the cubulin crystal structure (PDB: 3KQ4). **(B)** MFRP LDLA1 domain modeled off LDLR structure (PDB: 3P5B). **(C)** MFRP CUB2 domain modeled off the cubulin crystal structure (PDB: 3KQ4). **(D)** MFRP LDLA2 domain modeled off LDLR structure (PDB: 3P5B). **(E)** MFRP FZ-CRD domain modeled off the XWnt8-bound Frizzled-8 structure (PDB: 4F0A) as a template. The individual domains were joined using the homology-modeling protocol in the YASARA 15.7.25 software package to generate a MFRP model. The model was refined with an energy minimization in the YAMBER3 force field followed by a steepest descent minimization and simulated annealing.

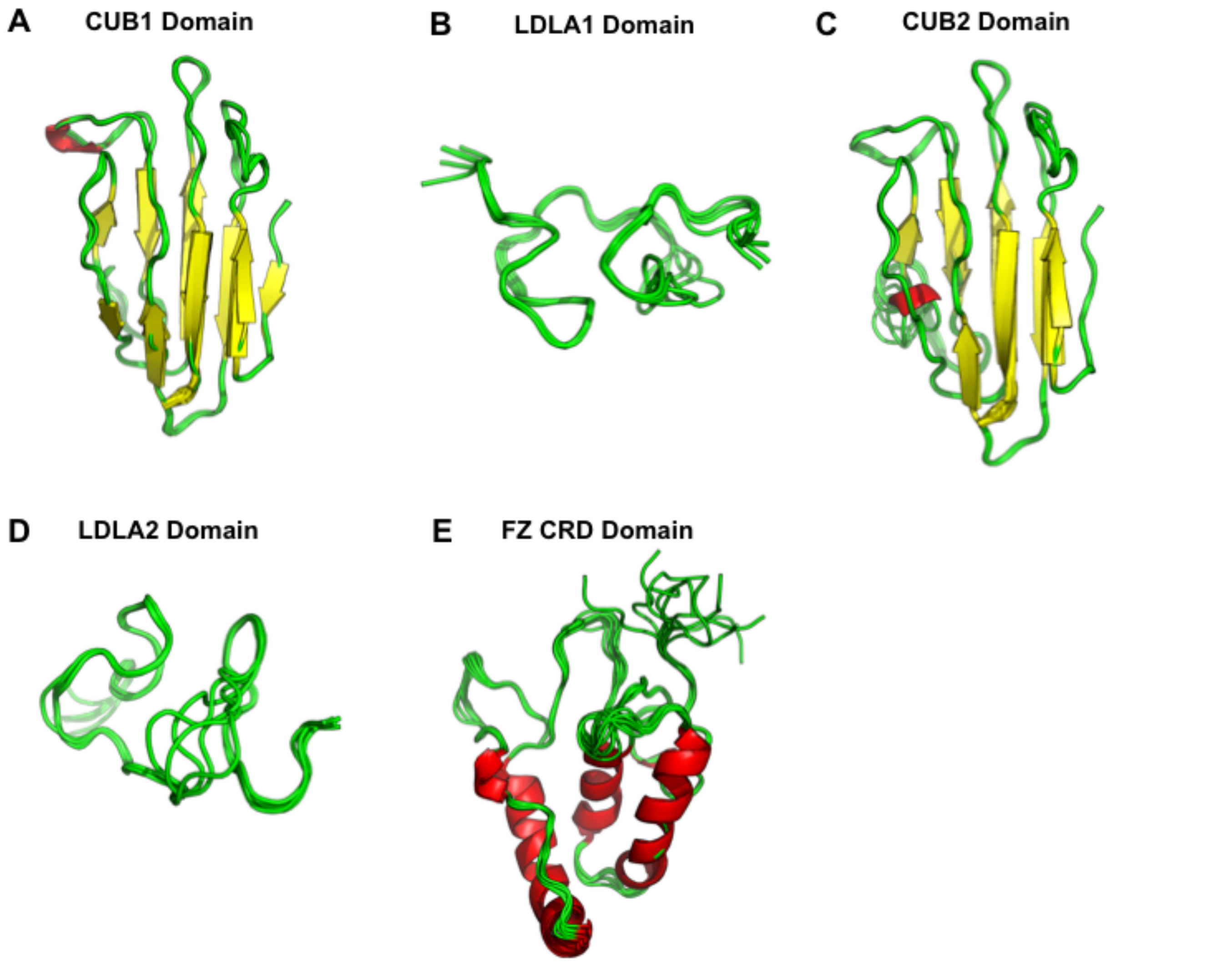

Supplemental Figure 3. Structural modeling of *MFRP* patient mutations. (A) Schematic representation of patient mutations on the secondary structure of MFRP. The *rd6* mutation expresses in a truncated protein. The p.His384Profs*8 mutation leads to an insertion of a proline in the CUB2 domain followed by a frameshift. The p.Arg539Cys mutation adds an additional cysteine to the Frizzled cysteine-rich domain (CRD) of MFRP. The IVS10, +5, G>A is a splice donor mutation and does not affect the secondary structure. Finally, the c.492delC mutant expresses a truncated protein. **(B)** Homology-based model of human MFRP. **(C)** Predicted location of known mutations on MFRP structure. Mutations are found throughout multiple domains and are therefore predicted to affect multiple functions of the protein.

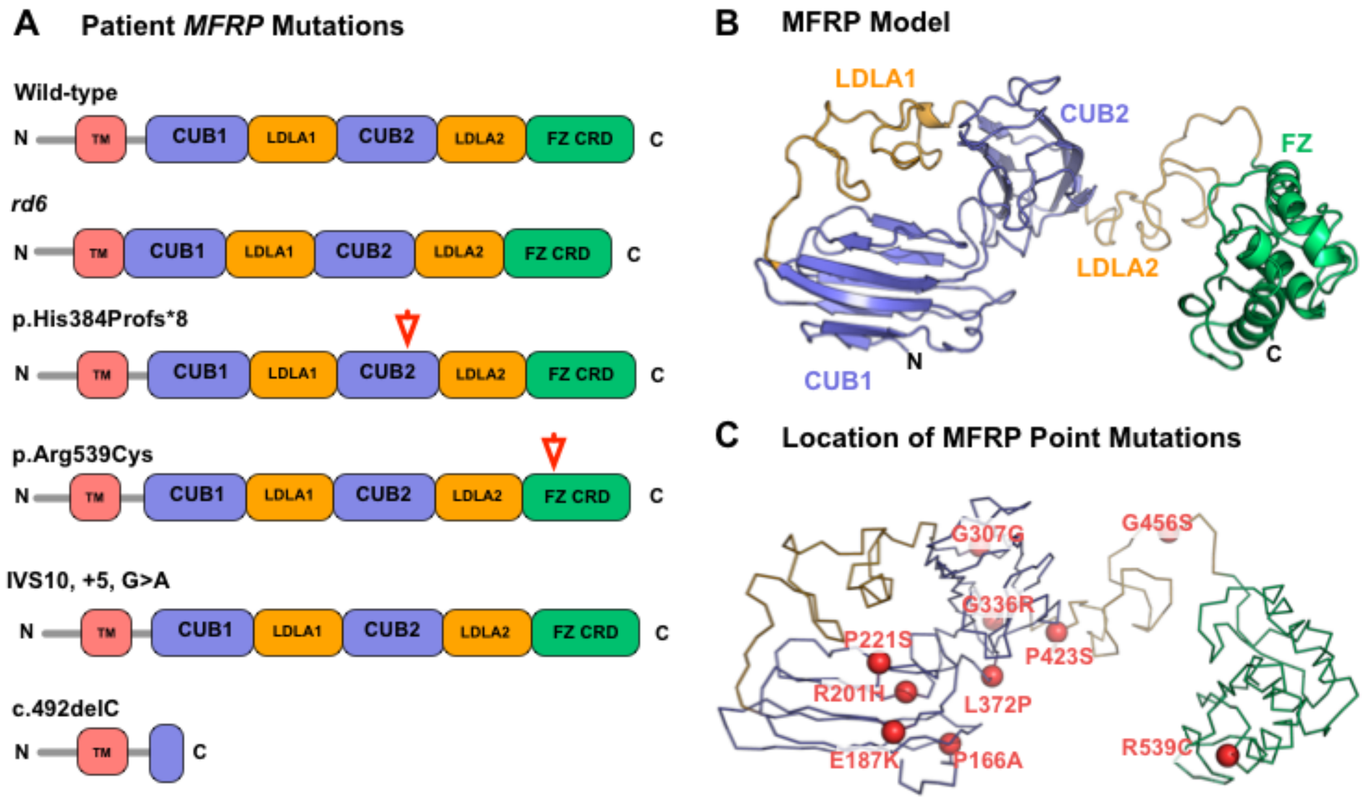

Supplemental Figure 4. RPE-choroid proteomic analysis pipeline. (A) The RPE-choroid were dissected from 9 mouse eyes. **(B)** Proteins were precipitated from dissected RPE-choroid using chloroform methanol and dissolved using 0.1% Rapidest detergent in 50 mM ammonium bicarbonate. Proteins were digested with trypsin. **(C)** Peptide intensities were analyzed by liquid chromatography-tandem mass spectrometry. Three biological replicates were analyzed using a Synapt G2 quadrupole-time-of-flight mass spectrometry (QTOF; Waters Corporation, Milford, MA). **(D)** The data were analyzed with MS^E/$Identity^E$ algorithm (PLGS software Version 2.5 RC9) and Rosetta Elucidator software. Elucidator software detected 383,353 features across 27 LC/MS runs. Identifications were returned on 3,132 proteins with a PLGS score > 300 (pass 1 data only) and 4% false discovery rate. **(E)** Of the 3,132 proteins, 2,089 were represented by two or more peptides and used in further differential expression, ontology, and pathway analysis. Illustrations by Lucy Evans and Vinit Mahajan (acknowledgements section).

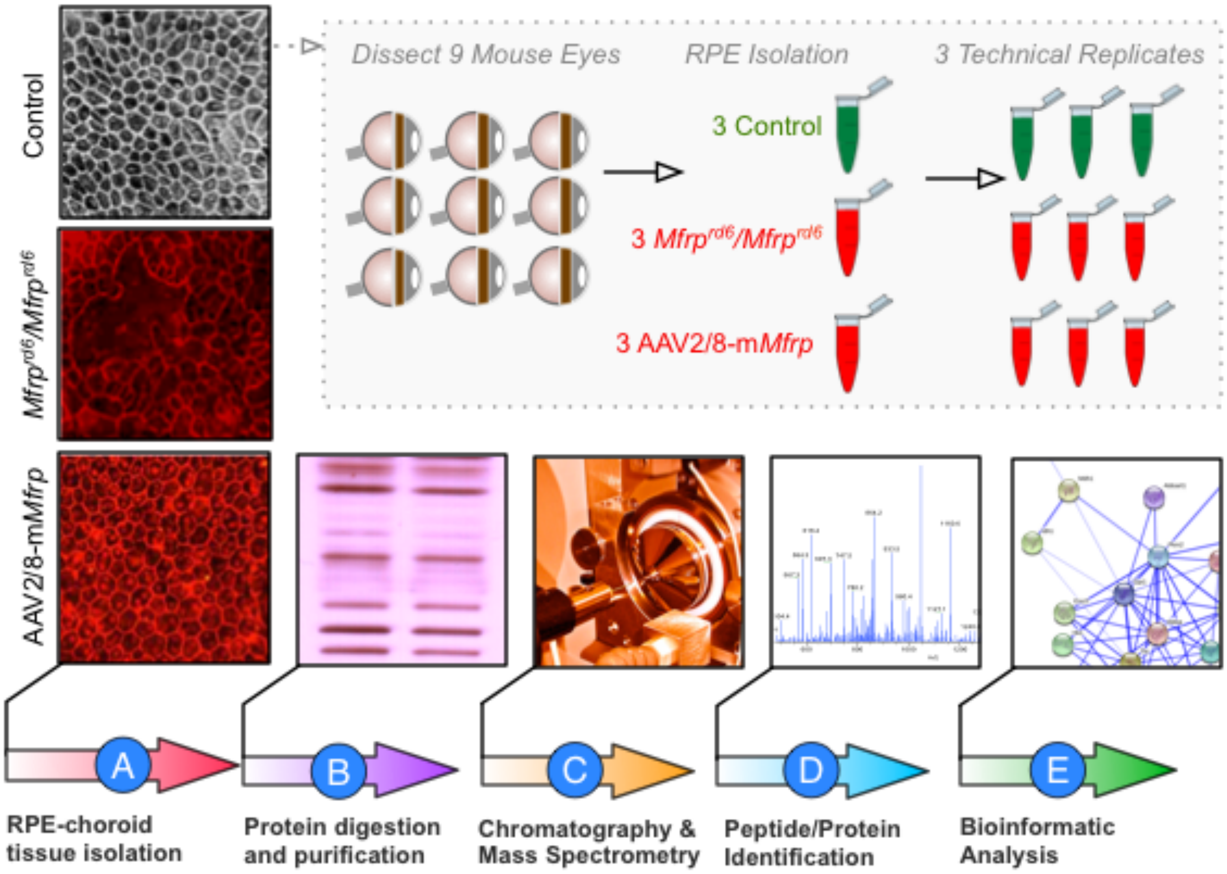

Supplemental Figure 5. Pathway analysis. Proteins in this list were interrogated using pathway analysis. The top 10 pathways are represented and are ranked by their log (p-value), obtained from the right-tailed Fisher Exact Test, and by their ratio of enrichment, which is equal to the number of observed proteins divided by the number of expected proteins from each pathway that is represented. **(A)** Proteins upregulated in *Mfrp*^{rd6}/*Mfrp*^{rd6} mice compared to controls. These proteins were restored to control levels following gene therapy ($p<0.05$). **(B)** A total of 36 proteins were upregulated in AAV2/8-m*Mfrp* mice that were not seen in control mice or in *Mfrp*^{rd6}/*Mfrp*^{rd6} mice. We hypothesized that these pathways were upregulated in response to the AAV vector injection ($p<0.05$). **(C)** A total of 12 proteins were restored from *Mfrp*^{rd6}/*Mfrp*^{rd6} levels following gene therapy ($p<0.05$). Details of the individual proteins are listed in Table S4. **(D)** A total of 65 proteins were not rescued to control levels following gene therapy.

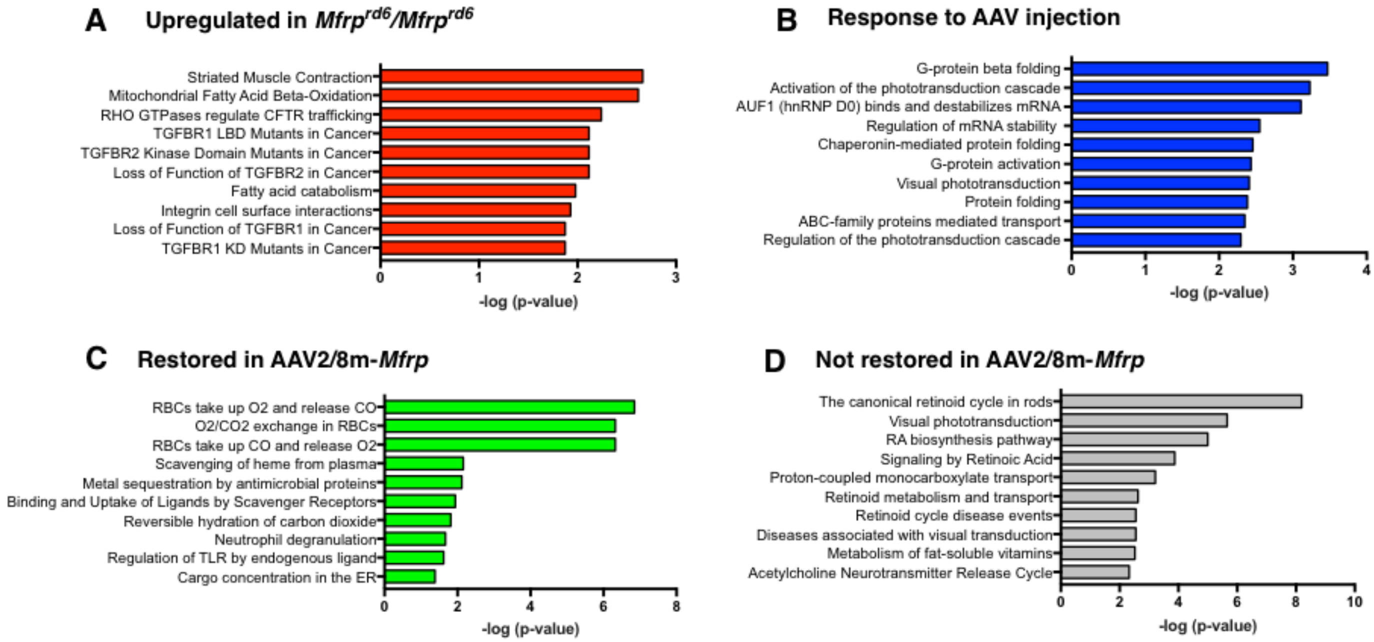

Table S1. *MFRP* point Mutations

Mutation	Amino acid change	Affected Domain
c.760G>A	Arg54Gly	Transmembrane
c.496C>G	Pro166Ala	CUB1
c.559G>A	Glu187Lys	CUB1
c.602G>A	Arg201His	CUB1
c.661C>T	Pro221Ser	CUB1
c.664C>A	Pro222Thr	CUB1
c.919G>A	Gly307Cys	CUB2
c.1006G>A	Gly336Arg	CUB2
c.1115T>C	Leu372Pro	CUB2
c.1267C>T	Pro423Ser	CUB2
c.1366G>A	Gly456Ser	LDL2
c.1542G>C	Gln514His	FZD/CRD
c.1549C>T	Arg517Trp	FZD/CRD
c.1615C>T	Arg539Cys	FZD/CRD

Table S2. Proteins upregulated in *Mfrp*^{rd6}/*Mfrp*^{rd6} mice.

Uniprot ID	Uniprot name	Protein names
O54983	CRYM_MOUSE	Ketimine reductase mu-crystallin (EC 1.5.1.25) (NADP-regulated thyroid-hormone-binding protein)
P52196	THTR_MOUSE	Thiosulfate sulfurtransferase (EC 2.8.1.1) (Rhodanese)
Q6GQT1	A2MG_MOUSE	Alpha-2-macroglobulin-P (Alpha-2-macroglobulin)
Q60634	FLOT2_MOUSE	Flotillin-2 (Membrane component chromosome 17 surface marker 1 homolog)
Q6ZWY3	RS27L_MOUSE	40S ribosomal protein S27-like
Q8R527	RHOQ_MOUSE	Rho-related GTP-binding protein RhoQ (Ras-like protein TC10)
O88200	CLC11_MOUSE	C-type lectin domain family 11 member A
Q9R0G6	COMP_MOUSE	Cartilage oligomeric matrix protein (COMP)
Q80YQ1	Q80YQ1_MOUSE	Thrombospondin 1
P09542	MYL3_MOUSE	Myosin light chain 3
Q9QYR9	ACOT2_MOUSE	Acyl-coenzyme A thioesterase 2, mitochondrial (Acyl-CoA thioesterase 2)
Q9CPP6	NDUA5_MOUSE	NADH dehydrogenase [ubiquinone] 1 alpha subcomplex subunit 5
P33267	CP2F2_MOUSE	Cytochrome P450 2F2 (Naphthalene hydroxylase)
P32848	PRVA_MOUSE	Parvalbumin alpha
P20801	TNNC2_MOUSE	Troponin C, skeletal muscle (STNC)
Q3U9N4	Q3U9N4_MOUSE	Granulins (Putative uncharacterized protein)
P26883	FKB1A_MOUSE	Peptidyl-prolyl cis-trans isomerase FKBP1A (Rotamase)
P16460	ASSY_MOUSE	Argininosuccinate synthase (EC 6.3.4.5) (Citrulline--aspartate ligase)
P09528	FRIH_MOUSE	Ferritin heavy chain (Ferritin H subunit) (EC 1.16.3.1
P27546	MAP4_MOUSE	Microtubule-associated protein 4 (MAP-4)

Table S3. Proteins upregulated as a response to AAV2 injection

Uniprot ID	Uniprot name	Protein names
P22599	A1AT2_MOUSE	Alpha-1-antitrypsin 1-2 (AAT)
F6UXV2	F6UXV2_MOUSE	Serine-protein kinase ATM (Fragment)
Q00623	APOA1_MOUSE	Apolipoprotein A-I
P49722	PSA2_MOUSE	Proteasome subunit alpha type-2
A2AE89	A2AE89_MOUSE	Glutathione S-transferase Mu 1
A2AL18	A2AL18_MOUSE	Rho GTPase-activating protein 11A
P15409	OPSD_MOUSE	Rhodopsin
P20612	GNAT1_MOUSE	Guanine nucleotide-binding protein G(t) subunit alpha-1 (Transducin alpha-1 chain)
P17879	HS71B_MOUSE	Heat shock 70 kDa protein 1B (Heat shock 70 kDa protein 1) (HSP70.1)
P45952	ACADM_MOUSE	Medium-chain specific acyl-CoA dehydrogenase, mitochondrial (MCAD) (EC 1.3.8.7)
Q61133	GSTT2_MOUSE	Glutathione S-transferase theta-2 (EC 2.5.1.18) (GST class-theta-2)
Q9D8U8	SNX5_MOUSE	Sorting nexin-5
Q60847	COCA1_MOUSE	Collagen alpha-1(XII) chain
Q9CQG9	TM100_MOUSE	Transmembrane protein 100
Q9Z1Q9	SYVC_MOUSE	Valine--tRNA ligase (EC 6.1.1.9) (Protein G7a) (Valyl-tRNA synthetase) (ValRS)
B1AZA7	B1AZA7_MOUSE	Alpha-catulin
Q61029	LAP2B_MOUSE	Lamina-associated polypeptide 2
P80315	TCPD_MOUSE	T-complex protein 1 subunit delta (TCP-1-delta) (A45) (CCT-delta)
Q8BG05	ROA3_MOUSE	Heterogeneous nuclear ribonucleoprotein A3 (hnRNP A3)
P63005	LIS1_MOUSE	Platelet-activating factor acetylhydrolase IB subunit alpha)
B2RSH2	GNAI1_MOUSE	Guanine nucleotide-binding protein G(i) subunit alpha-1
Q8VDW0	DX39A_MOUSE	ATP-dependent RNA helicase DDX39A
Q9D552	SPT17_MOUSE	Spermatogenesis-associated protein 17
Q80X68	Q80X68_MOUSE	Citrate synthase
Q922J9	FACR1_MOUSE	Fatty acyl-CoA reductase 1 (EC 1.2.1.n2)
P13634	CAH1_MOUSE	Carbonic anhydrase 1
P83882	RL36A_MOUSE	60S ribosomal protein L36a (60S ribosomal protein L44)
Q8C726	BTBD9_MOUSE	BTB/POZ domain-containing protein 9
Q3UIU2	NDUB6_MOUSE	NADH dehydrogenase [ubiquinone] 1 beta subcomplex subunit 6
Q6PD31	TRAK1_MOUSE	Trafficking kinesin-binding protein 1
P51885	LUM_MOUSE	Lumican (Keratan sulfate proteoglycan lumican) (KSPG lumican)
Q9CPQ8	ATP5L_MOUSE	ATP synthase subunit g, mitochondrial
O88685	PRS6A_MOUSE	26S protease regulatory subunit 6A
P24472	GSTA4_MOUSE	Glutathione S-transferase A4

Table S4. Proteins rescued by gene therapy

Uniprot ID	Uniprot name	Protein names
Q00897	A1AT4_MOUSE	Alpha-1-antitrypsin 1-4
Q91VB8	Q91VB8_MOUSE	Alpha globin
P01942	HBA_MOUSE	Hemoglobin subunit alpha
F6ZFS0	F6ZFS0_MOUSE	EGF-containing fibulin-like extracellular matrix protein 1
A8DUK4	A8DUK4_MOUSE	Beta-globin
Q61702	ITIH1_MOUSE	Inter-alpha-trypsin inhibitor heavy chain H1
D3YUE2	D3YUE2_MOUSE	Procollagen C-endopeptidase enhancer 1
Q91WQ3	SYYC_MOUSE	Tyrosine--tRNA ligase
Q9DCS9	NDUBA_MOUSE	NADH dehydrogenase [ubiquinone] 1 beta subcomplex subunit 10
F8WHM5	F8WHM5_MOUSE	Golgi apparatus protein 1 (Fragment)
Q9CZ62	CEP97_MOUSE	Centrosomal protein of 97 kDa (Cep97)
P00920	CAH2_MOUSE	Carbonic anhydrase 2
Q8K480	MFRP_MOUSE	Membrane frizzled-related protein
P31725	S10A9_MOUSE	Protein S100-A9

Table S5. Proteins not rescued by gene therapy

Uniprot ID	Uniprot name	Protein names
P46425	GSTP2_MOUSE	Glutathione S-transferase P 2
P04344	CRGB_MOUSE	Gamma-crystallin B
D3YX34	D3YX34_MOUSE	Dynactin subunit 1
Q6VYI4	Q6VYI4_MOUSE	TAR DNA-binding protein 43
P24622	CRYAA_MOUSE	Alpha-crystallin A chain
Q9QZF2	GPC1_MOUSE	Glypican-1
G3UZI2	G3UZI2_MOUSE	Heterogeneous nuclear ribonucleoprotein Q
Q62417	SRBS1_MOUSE	Sorbin and SH3 domain-containing protein 1
Q9CZ44	NSF1C_MOUSE	NSFL1 cofactor p47 (p97 cofactor p47)
P17710	HXK1_MOUSE	Hexokinase-1
Q91WJ8	FUBP1_MOUSE	Far upstream element-binding protein 1 (FBP)
O08599	STXB1_MOUSE	Syntaxin-binding protein 1
P09405	NUCL_MOUSE	Nucleolin (Protein C23)
P56546	CTBP2_MOUSE	C-terminal-binding protein 2 (CtBP2)
O35945	AL1A7_MOUSE	Aldehyde dehydrogenase, cytosolic 1
Q62148	AL1A2_MOUSE	Retinal dehydrogenase 2
P63011	RAB3A_MOUSE	Ras-related protein Rab-3A
P15105	GLNA_MOUSE	Glutamine synthetase
Q99JR1	SFXN1_MOUSE	Sideroflexin-1
Q80U72	SCRIB_MOUSE	Protein scribble homolog (Scribble) (Protein LAP4)
E9Q1Z0	E9Q1Z0_MOUSE	Keratin 90
P11438	LAMP1_MOUSE	Lysosome-associated membrane glycoprotein 1
Q8C9S4	CC186_MOUSE	Coiled-coil domain-containing protein 186
H3BLG5	H3BLG5_MOUSE	Syntenin-1 (Fragment)
Q8VCH7	RDH10_MOUSE	Retinol dehydrogenase 10
Q61171	PRDX2_MOUSE	Peroxiredoxin-2
Q61553	FSCN1_MOUSE	Fascin
O54984	ASNA_MOUSE	ATPase Asna1
E9Q2W9	E9Q2W9_MOUSE	Alpha-actinin-4
O55240	RDH1_MOUSE	11-cis retinol dehydrogenase
P61089	UBE2N_MOUSE	Ubiquitin-conjugating enzyme E2 N
Q61990	PCBP2_MOUSE	Poly(rC)-binding protein 2
Q9CQ19	MYL9_MOUSE	Myosin regulatory light polypeptide 9
Q62132	PTPRR_MOUSE	Receptor-type tyrosine-protein phosphatase R

Q71M36	CSPG5_MOUSE	Chondroitin sulfate proteoglycan 5
P97427	DPYL1_MOUSE	Dihydropyrimidinase-related protein 1
P21956	MFGM_MOUSE	Lactadherin (MFGM)
H3BL49	H3BL49_MOUSE	T-complex protein 1 subunit theta
O35308	MOT3_MOUSE	Monocarboxylate transporter 3
Q8BMF3	MAON_MOUSE	NADP-dependent malic enzyme, mitochondrial
Q80TB8	VAT1L_MOUSE	Synaptic vesicle membrane protein VAT-1 homolog-like
Q9QXC6	Q9QXC6_MOUSE	Beta-A3/A1 crystallin protein
Q60737	CSK21_MOUSE	Casein kinase II subunit alpha
P43275	H11_MOUSE	Histone H1.1 (H1 VAR.3)
P29812	TYRP2_MOUSE	L-dopachrome tautomerase
P18572	BASI_MOUSE	Basigin
Q9D0M5	DYL2_MOUSE	Dynein light chain 2, cytoplasmic
P62696	CRBB2_MOUSE	Beta-crystallin B2
Q9Z275	RLBP1_MOUSE	Retinaldehyde-binding protein 1
Q91ZQ5	RPE65_MOUSE	Retinoid isomerohydrolase
Q00915	RET1_MOUSE	Retinol-binding protein 1
Q99M71	EPDR1_MOUSE	Mammalian ependymin-related protein 1
Q9JI60	LRAT_MOUSE	Lecithin retinol acyltransferase
Q91XU3	PI42C_MOUSE	Phosphatidylinositol 5-phosphate 4-kinase type-2 gamma
Q9Z0P5	TWF2_MOUSE	Twinfilin-2 (A6-related protein)
P19246	NFH_MOUSE	Neurofilament heavy polypeptide (NF-H)
E9Q4P0	E9Q4P0_MOUSE	KxDL motif-containing protein 1)
F2Z468	F2Z468_MOUSE	Matrilin-4
Q9EQ80	NIF3L_MOUSE	NIF3-like protein 1
Q9Z1T2	TSP4_MOUSE	Thrombospondin-4
Q505F5	LRC47_MOUSE	Leucine-rich repeat-containing protein 47
Q9R0H0	ACOX1_MOUSE	Peroxisomal acyl-coenzyme A oxidase 1 (AOX)
P50171	DHB8_MOUSE	Estradiol 17-beta-dehydrogenase 8
P47964	RL36_MOUSE	60S ribosomal protein L36
A2ATI9	A2ATI9_MOUSE	Golgi reassembly stacking protein 2, isoform CRA_d
